# Optimisation of the machining time required by insole orthotic shoes for patients with clubfoot using the Taguchi and response surface methodology approach

**DOI:** 10.1016/j.heliyon.2023.e16860

**Published:** 2023-06-01

**Authors:** P.W. Anggoro, B. Bawono, D.B. Setyohadi, L. Ratnasari, P.K. Fergiawan, M. Tauviqirrahman, J. Jamari, A.P. Bayuseno

**Affiliations:** a Department of Industrial Engineering, University of Atma Jaya Yogyakarta, Jl. Babarsari 44, Yogyakarta, 55281, Indonesia; b Department Informatics, University of Atma Jaya Yogyakarta, Jl Babarsari 44, Yogyakarta, 55281, Indonesia; c Department of Science and Mathematics, University of Diponegoro, Jl. Prof. Soedarto, SH., Tembalang, Semarang, 50275, Indonesia; d Department of Mechanical Engineering, University of Diponegoro, Jl. Prof. Soedarto, SH., Tembalang, Semarang, 50275, Indonesia

**Keywords:** Insole, CARE system, Taguchi method (TM), Response surface methodology (RSM), Desirability function (DF), Machining time (t_m_)

## Abstract

In this study, the application of the computer-aided reverse engineering system (CARE) to the novel design and manufacture of a comfortable insole for a clubfoot patient is presented. The Taguchi method (TM) and response surface methodology (RMS) were used to predict the machining time of the orthotic boot insole during both computer-aided manufacturing (CAM) simulation and computer numerical control (CNC) machining. Taguchi’s experimental design, presented as a matrix orthogonal array L_27_3^6^, was acquired for controlling parameters, namely tool path strategy (A), spindle speed (B), step-down (C), step-over of the cutter (D), cutter diameter (E), and dimensional tolerance (F) of the insole size. In this method, the model generated by the RMS method evaluates the six parameters influencing the machining time. The objective of this study is to develop a regression model that demonstrates the relationship between the cutting parameters and insole machining time. The optimal parameters are A_1_B_1_C_3_D_2_E_1_F_2_, where A_1_ denotes raster finishing, B_1_ denotes a spindle speed of 10,000 rpm, C_3_ denotes a step-down of 850 mm, D_2_ denotes a step-over of 0.25 mm, E_1_ denotes a cutter diameter of 20–35 mm, and F_2_ deontes a tolerance of 0.75 mm. The experimental and calculated machining time (t_m_) results were 236 and 125.4 min, respectively. However, the real machining results were 334 and 152.25 min with error values of 46.86% and 54.42%, respectively. Meanwhile, with the t_m_ RMS method, the simulated and calculated machining time results were 189.22 and 236.35 min, whereas the real t_m_ values were 236.52 and 334.86 min with error values of 19.94% and 29.37%, respectively. This research obtains improvements of 19.82% (simulation time) and 29.19% (real-time).

## 1 Introduction

The shoe industry is growing owing to advancements in product design and innovations is fabrication processess [1,2]. However, this ongoing process mainly considers the fabrication of regular and fashioned shoes. Conversely, there are only limited customized shoe products in the market designed for patients with the clubfoot defect. The main factor may be related to the limitation of traditional practices, in which these specific shoes are handmade to ensure the appropriate size fit for patients with the clubfoot defect. Remarkably, the specifically shaped shoes of patients require specific shoe insole orthotics to ensure comfortable use [3].

The production process of the insoles for deformed feet requires reverse engineering (RE) technology based on computer-aided design (CAD), computer-aided manufacturing (CAM), and reliable computer numerical control (CNC). The combined strategy is called the computer-aided reverse engineering (CARE) system [[4], [5], [6], [7], [8], [9]]. Accordingly, considerable efforts on RE has been incorporated into product design [[6], [7], [8], [9], [10], [11]]. RE is crucial in the design of many shoe types. Specifically, RE designs can be used to modify existing products. The CARE system has been widely implemented in footwear manufacturing [[3], [4], [5], [6], [7], [8]]. It has been previously demonstrated that machining orthotics or ankle-foot orthotic (AFO) footwear with a CNC machine yielded orthotic products with accurate and precise dimensions according to the patient's foot shape [3,8]. Consequently, a combination of machining methodologies for fabricating insole products increased the precision and accuracy of AFO products to the desired orthopaedic standard [4].

Product development may include stages ranging from physical to digital mockups and 2D to 3D designs. CAD technology has been fully implemented in the digital product design processes. Modelling uses several computer software types (Power SHAPE, ArtCAM, Toolmaker, Copy CAD, PS Moldmaker, and OrthoCAD), whereas tools for machining simulations may use PowerMILL, CAM feature, Art CAM, and Ortho Mill software (Delcam). To develop rapid 3D data acquisition systems, reverse engineering (RE) technology has become an essential tool in the product design community [1].

Currently, the designing insoles with CAD technology has reduced the product design time, thereby reducing product development costs [5,7]. CAD applications can help designers create footwear designs quickly, precisely, realistically, and consistently [6]. Specifically, CAD software facilitates the design of 3D insole models leading to a shift in product development from native to digital models or from 2D drawing designs to 2.5D or 3D drawing models for subsequent CNC machining [1,3,6,8,9].

Further application of CNC in the orthotics industry has increased productivity, production flexibility, and integration of system automation [12,13]. The CNC machining process has been reported to yield an insole product with good size accuracy and reduced production waste [14,15], as well as reduced machining time and product manufacturing costs [[17], [18], [19]]. CNC machines have long been used in the carpentry industry for sanding, milling, and drilling [20,21] with high spindle speeds of up to 60,000 rpm. This machining characteristic provides efficiency, accuracy, and quality when manufacturing insole products compared with conventional cutting practices [10]. In particular, the machining time can be optimized using an optimal and practical production plan [11]. Gavril et al. (2016) reported that an increase in productivity and cost optimisation in CNC manufacturing might reduce the working time by 32.49% and manufacturing costs by up to 10.33%.

Specifically, the machining time may be reduced by adjusting the cutting parameters in a CNC machine, including feed rate, step-over, tool radius, spindle speed, and depth of cut [[22], [23], [24]]. Selecting reasonable machining parameters may help engineers and researchers optimise the insole cutting process using CNC machines. In some cases, optimal machining strategies may be adopted based on CAM simulations, by which the optimized machining time can be predicted [3,[7], [8], [9]]. Optimising machining parameters may include adjusting the spindle speed, step-over, step-down, and tool path strategy [3,7,8,[13], [14], [15],^25^]. Here, an analytical approach is proposed to determine the minimum number of machining experiments at lower costs [26].

Therefore, the design of experiment (DoE) plays a key role in the methodology of engineering problems by expressing the factorial levels required for optimising the design and manufacturing of a product [[27], [28], [29], [30]]. A complete factorial design determines any possible combination of observed factor levels based on two or more factors [32]. Several previous studies [7,8,25,25,29,30,33] demonstrated that the DoE of machining parameters (i.e., depth of cut, rotation speed, and feed speed) using the Taguchi (TM) design and response surface method (RSM) could significantly minimise the surface roughness of customised insole products [7,25,33,[41], [42], [43]]. Similarly, CNC machining parameters, such as feed rate, spindle speed, and cutting speed, can be adjusted to reduce machining time [34]. Accordingly, a faster machining time may be attributed to higher feed rates and spindle speeds.

However, the use of TM and RSM for optimising drilling parameters (i.e., cutting tool diameter, spindle speed, and feed rate) suggested that the feed rate was the most significant factor influencing the machining time [35]. Similarly, the effect of tool path strategy, spindle speed, and feed rate on the CNC machining insole was examined for material-based ethylene-vinly acetate (EVA) foam rubber [8,25,33,41]. These parameters can be optimized using the Taguchi and RSM methods, minimising surface roughness of the insole rubber [25,28,33,36,[41], [42], [43]]. Other research were conducted on Beech, Walnut wood with RSM, and stone–plastic composite materials using the finite element method [[45], [46], [47]] (and produce optimal machining parameters). However, the control of machining parameters in CNC milling machines based on the Taguchi and RSM approaches for machining the insole rubber for patients with the clubfoot defect has not yet been researched.

The novelty of this study is that it combines traditional and modern methods to create better designs and optimise the fabrication of orthotic insole products for the clubfoot defect using the Taguchi and RSM approaches. This study systematically varied the machining parameters (i.e., tool path strategy, spindle speed, step-down, step-over of the cutter, cutter diameter, and dimensional tolerance of the insole size) to minimise the machining time. Based on the experimental design, the machining parameters affecting the production time of the insoles were examined separately, and the product comfort related to each paramenter was investigated under experimental conditions. Our results are expected to contribute to the novel design and manufacture of insole boots shoes.

## 2 Material and method

### 2.1 3D CAD modelling of insole boots shoes for the clubfoot defect

**Fig. 1:**
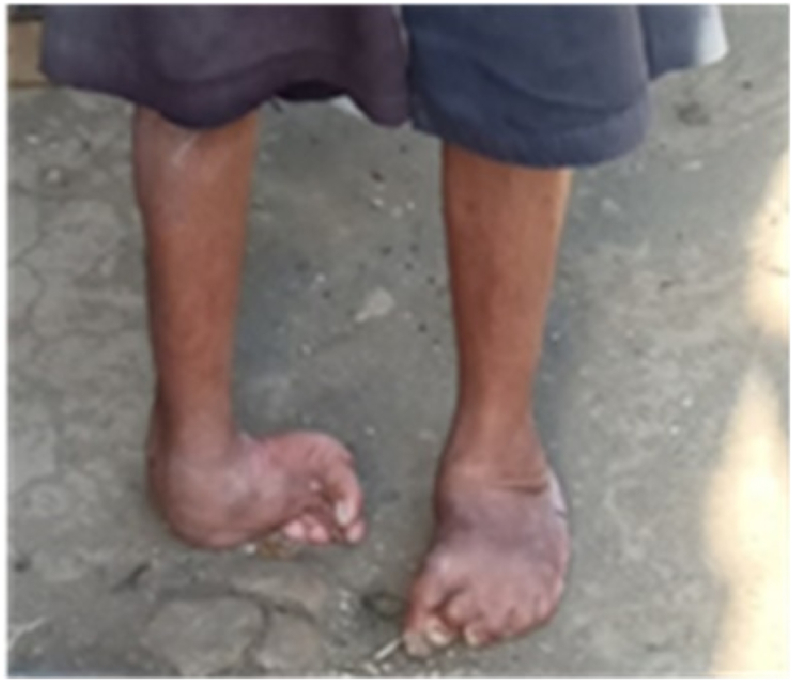
Patient with the clubfoot defect.

**Fig. 2:**
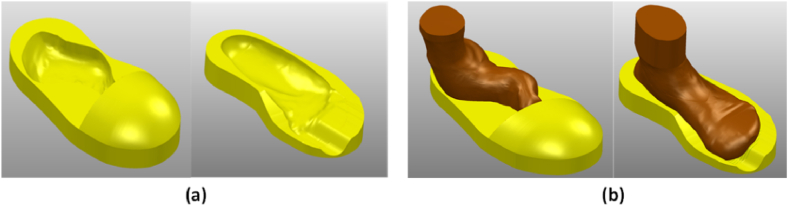
3D Modelled CAD Insole: (a) modelled insole shoes for the clubfoot patient, (b) 3D model assembly of the shoelast and insole.

**Fig. 3:**
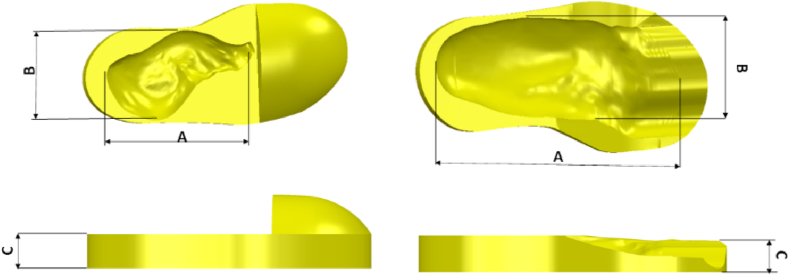
An enlarged tolerance scale for the insole: A = Length of ISO; B = Witdh of ISO; C = Height of ISO.

In this study, the CARE system technology was used as the first step to create a 3D duplicate of the limb out of the gypsum, which was then scanned using a scan tool [38]. The proposed technique is more accurate than the traditional foam box method [8]. For the moulding process, white and blue liquid gypsums were poured into a box to create a model of the patient’s foot (Fig. 1). A (Scan 700TM) scanning tool was then used to create the 3D CAD models of the patient’s legs in an *.STL format. Using CAD (PowerShape, 2019e) to generate the insole model yielded detailed leg contour lines in the *.STL data input. The curve-based model (CBS-Model) technique was established previously [3,38] and implemented to create 3D models of the feet and shoe insole designs. The insole design was constructed by extending the geometric scale (length and width) of the insole based on previously determined tolerances [3,[7], [8], [9],^38^]. The results are shown in Fig. 2, Fig. 3, with corresponding data reported in Table 1.

**Table 1:** 3D CAD model size enlargement of the orthotic insole boots shoe.

Tolerance (MM)	Left Clubfoot Patient Without Added Material			Right Clubfoot Patient Without Added Material		
	Dimension (mm)			Dimension (mm)		
	A = Length of ISO	B = Width of ISO	C = Height of ISO	Length of ISO (A)	Width of ISO (B)	Height of ISO (C)
0	245.94	109.13	35.03	172.91	97.22	42.42
0.75	246.69	110.14	35.03	173.48	98.06	42.42
1.5	247.44	111.65	35.03	175.58	99.85	42.42

### 2.2 Machining condition and experimental design

During the experimental CNC milling, six parameters were varied to control the machining time, namely, the tool path (factor A), spindle speed (factor B), step-down (factor C), step-over (factor D), cutter diameter (factor E), and insole size tolerance (factor F). The machining parameter levels were determined based on the machining times listed in Table 2. The Taguchi orthogonal array (OA) with six parameters and two levels (L_27_3^6^) is summarised in Table 3.

**Table 2:** Machining parameters.

Code	Factor	Level		
		1	2	3
A	Tool path	Raster Finishing	Optimized Constants Z	Step and Shallow
B	Spindle Speed (rpm)	10,000	13,000	15,000
C	Stepdown (mm)	5	3	2
D	Stepover (mm)	4	3	2
E	Diameter Cutter (mm)	6	4	2
F	Tolerance (mm)	0	0.75	1.5

**Table 3:** Design matrix OA L_27_3^6^.

No	Actual Values						Simulation time (min)	Machining time (min)
	Tool path	Spindle Speed	Step-Down	Step-Over	Cutter	Tolerance		
1	Raster Finishing	10,000	5	4	6	0	160	200
2	**Raster Finishing**	**10,000**	**5**	**4**	**4**	**0.75**	**158**	**209**
3	Raster Finishing	10,000	5	4	2	1.5	156	233
4	Raster Finishing	13,000	3	3	6	0	150	188
5	Raster Finishing	13,000	3	3	4	0.75	158	198
6	Raster Finishing	13,000	3	3	2	1.5	195	244
7	Raster Finishing	15,000	2	2	6	0	152	190
8	Raster Finishing	15,000	2	2	4	0.75	162	203
9	Raster Finishing	15,000	2	2	2	1.5	188	235
10	Optimized Constants Z	10,000	3	2	6	0.75	151	189
11	Optimized Constants Z	10,000	3	2	4	1.5	201	251
12	Optimized Constants Z	10,000	3	2	2	0	231	289
13	Optimized Constants Z	13,000	2	4	6	0.75	172	215
14	Optimized Constants Z	13,000	2	3	4	1.5	183	229
15	Optimized Constants Z	13,000	2	2	2	0	194	243
16	Optimized Constants Z	15,000	5	4	6	0.75	179	224
17	Optimized Constants Z	15,000	5	3	4	1.5	168	210
18	Optimized Constants Z	15,000	5	2	2	0	183	229
19	Step and Shallow	10,000	2	4	6	1.5	181	226
20	Step and Shallow	10,000	2	3	4	0	192	240
21	Step and Shallow	10,000	2	2	2	0.75	215	269
22	Step and Shallow	13,000	5	4	6	1.5	211	264
23	Step and Shallow	13,000	5	3	4	0	219	274
24	Step and Shallow	13,000	5	2	2	0.75	232	290
25	Step and Shallow	15,000	3	4	6	1.5	244	305
26	Step and Shallow	15,000	3	3	4	0	252	315
27	Step and Shallow	15,000	3	2	2	0.75	269	336

In the Taguchi method, the signal-to-noise (S/N) ratio is used to assess the elements for the data response. When optimising the parameters, the S/N ratio is evaluated based on three characteristics: “the lowest is the best”, “the highest is the best”, and “the highest nominal is the best”. Accordingly, the S/N ratio can express the average simulation and machining time of insole boots shoes under ideal settings as follows [7,25,26,33,38]:

(1) $$SN\;Ratio=-10\;\log\;10\;\left(\left({y_{1}}^{2}+{y_{2}}^{2}+{y_{3}}^{2}+...+{y_{n}}^{2}\right)/n\right)$$

The data response of the machining time recorded during the CAM simulation and CNC machining processes under the test conditions are represented by variables Y_1_, Y_2_, Y_3_, …, and Y_n_. For the 27 experiments, the S/N Ratio calculated using Equation (1) provided both durations of machining time corresponding to the appropriate levels (Table 4, Table 5).

**Table 4:** Response value for S/N ratio (dB).

Factor	Simulation time (min)				Time real machine (min)			
	level 1	Level 2	Level 3	Delta	Level 1	Level 2	Level 3	Delta
	(dB)			(μm)	(dB)			(μm)
A	**168.7**	184.7	223.9	55.2	**210.8**	230.8	279.9	69.0
B	**187.1**	190.4	199.7	12.6	**233.9**	238.1	249.6	15.7
C	189.4	205.7	**182.1**	23.6	236.8	257.1	**227.6**	29.4
D	203.0	**180.1**	194.1	22.9	253.8	**225.1**	242.6	28.6
E	**177.8**	189.1	210.3	32.6	**222.2**	236.4	262.9	40.7
F	192.6	**189.4**	195.2	5.80	240.7	**236.8**	244.0	7.20

**Table 5:** Response value for means of effect.

Factor	Simulation time (min)				Time Real Machine (min)			
	level 1	Level 2	Level 3	Delta	Level 1	Level 2	Level 3	Delta
	(dB)			(μm)	(dB)			(μm)
A	**−44.5**	−45.27	−46.94	2.43	**−46.44**	−47.21	−48.88	2.43
B	**−45.37**	−45.51	−45.83	0.46	**−47.31**	−47.45	−47.77	0.46
C	−45.48	−46.06	**−45.17**	0.90	−47.42	−48	**−47.10**	0.90
D	−46.00	**−45.06**	−45.65	0.94	−47.94	**−47**	−47.59	0.94
E	**−44.89**	−45.44	−46.39	1.50	**−46.82**	47.38	−48.33	1.50
F	−45.56	**−45.39**	−45.76	0.37	−47.50	**47.33**	−47.70	0.37

### 2.3 Simulation of machining parameters

**Fig. 4:**
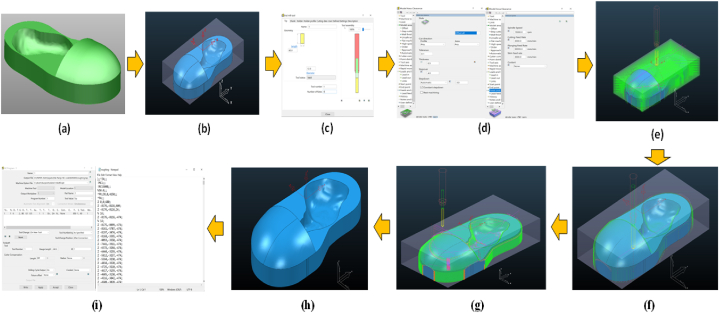
The stages of the CAM process methodology: (a) 3D CAD model of the clubfoot insole, (b) material size settings in PowerMill 2016, (c) cutter settings, (d) input parameters related to the tool path strategy, (e) tool path model area clearance (roughing), (f) tool path raster finishing (semi-finishing), (g) toolpath step and shallow (finishing), (h) simulation results of AFO products for patients with clubfoot foot deformities, (i) machining NC-Code.

In this study, the CAM software was used for the simulation to avoid manufacturing errors (trial and error). The final output of the CAM is NC-Code, which can be used to run programs on CNC machines. Using the CAM (PowerMill 2016) software for optimisation based on Taguchi simulation yielded the best 3D design of the clubfoot insole model [[7], [8], [9]]. The machining strategy for tool path selection is critical because it controls the machining time [7,9,16]. Correspondingly, the spindle speed, feed rate, depth of cut, cutter selection, and insole dimensions are the basic data for creating a tool path strategy. The response was determined to represent the machining time in the CAM simulation for each machining operation. Fig. 4(a–f) depicts the process of creating a tool path plan using the CAM software. The final product of the 3D CAD design was the CTEV shoe insole [see Fig. 2(a and b)], and a geometric variation of the product based on tolerances was established on the X and Y axes (Fig. 3 and Table 1). The optimal tool path strategy and machining time are listed in Table 2, Table 3).

### 2.4 Workpiece material, machine tools, and cutting tool specifications

EVA foam rubber was used as the insole material with dimensions of 300 × 200 × 50 mm and hardness level of 35–45 HRC (level 2) because it was employed previously [33,41,43] for shoe orthotics. Moreover, this material was selected because it is suitable as an insole material for orthotic boots [8,33,41]. Generally, EVA rubber with a size of 1200 × 2400 × 50 mm has a price range of $ 37.00/sheet, based on price data taken from the local market of Jakarta, Indonesia.

Furthermore, a 3D CAD model (Fig. 2) was run on the CAM for optimisation. This operation with the CNC machine was initiated by selecting the best machining parameters based on the six factors (Table 2). The OA L2736 design layout is generated using the Taguchi method and six factors. During the CAM simulation and use of CNC milling machines, 27 experimental treatments were required for the insole manufacturing process.

**Fig. 5:**
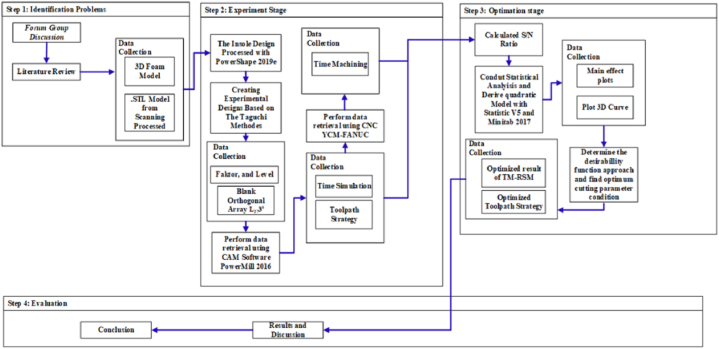
Flowchart of insole boots shoe production regarding the simulation time and machining time for optimisation of OA L_27_3^6^.

**Fig. 6:**
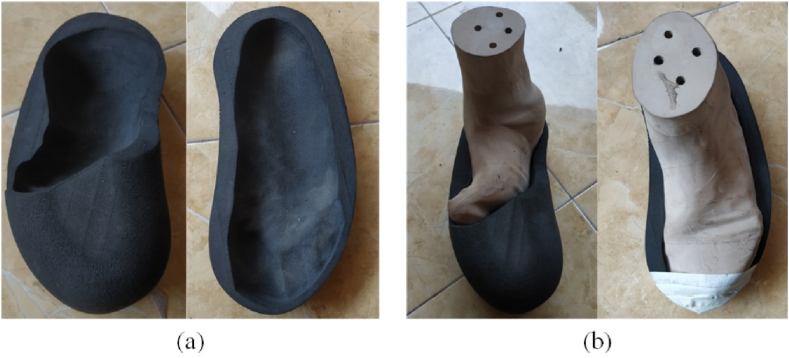
Component of boots shoe orthotic: (a) insole boots shoe for patient clubfoot, (b) insole with shoe last.

In the machining experiments of the insole boots shoes, a CNC milling machine (A YCM-Fanuc) with X-, Y-, and Z-axis travels of 1020, 600, and 600 mm, respectively, and maximum spindle speed of 15,000 rpm was selected. An end mill cutter [SECO, with specification 93060F] and ball-nose cutter [JS533060D1B0Z3-NXT] were the cutting tools utilised during the machining operation. The machining time simulated within the CAM program was calculated statistically, whereas the time during CNC machining was monitored using a timer through the roughing to finishing processes. Table 3 presents the recorded the CAM simulation and CNC machining times. Fig. 5 depicts the research stage, whereas Fig. 6(a and b) depicts the finished product.

For ethical clearance, the patients signed a consent form authorising the use of the information gathered during the scanning, design, production, and testing of the shoes. Prof. Dr. Banundari Rachmawati, Sp. PK (K), authorised the ethical approval according to Ethical Clearance No. 27/EC/KEPK/FK.UNDIP/II/2021, confirming that informed consent was obtained from all patients for our investigations.

## 3 Results and discussion

The participant, aged 65 years, had a congenital clubfoot deformity that was examined in the study (CTEV) (Fig. 1). Achondroplasia is one of the most prevalent congenital abnormalities affecting the musculoskeletal system [17]. Patients frequently experience difficulty in performing daily activities because of the shape of their legs. While standing, the right leg was twisted inward to a certain extent because the sole did not support the body. Meanwhile, the patient's left leg underwent a shift in bone position because of supporting the entire bodyweight of the patient when standing; therefore, this investigation focused on the patient's left leg.

In general, the foot-shape of a clubfoot patient does not fit the shape of the footwear, resulting in discomfort and even injury to the feet. In this case, the most important aspect for providing comfort is the proper design of the insole with correct-size-fitting shoes [2,3]. Correspondingly, the creation of a personalised insole for clubfoot patients starts with scanning and ends with an insole being manufactured on a CNC machine. The present work obtained the best insole product using a CNC machine and CAM-based production optimisation. Under the Taguchi approach, many manufacturing simulations have been conducted with various parameters. Accordingly, the orthogonal array (OA) L_27_3^6^ presented the best insole manufacturing design arrangement (Table 3). Fig. 3 and Table 1, Table 2 shows the initial settings input to the OA. The simulation and actual processing times correspond to the responses measured in this study (Table 3).

**Fig. 7:**
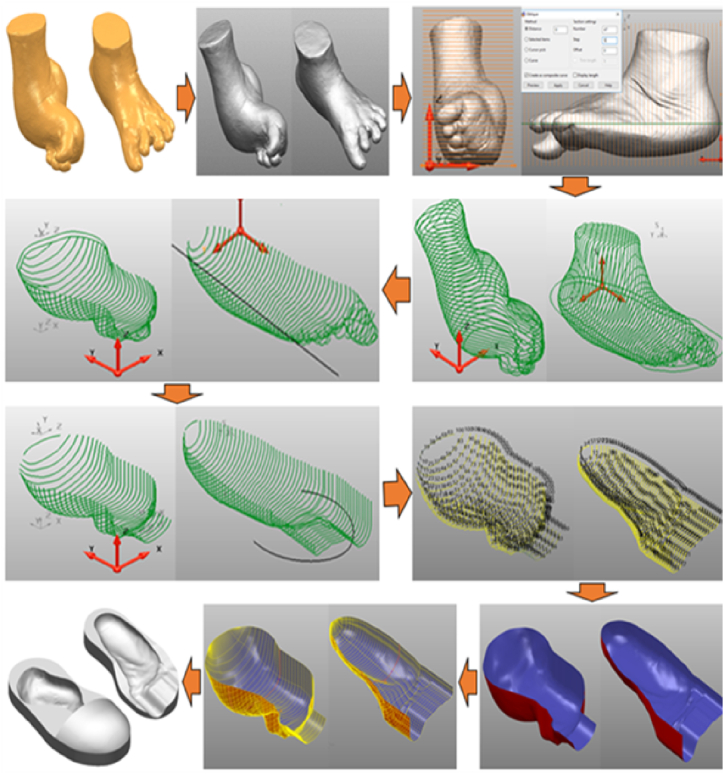
Application of CBS modelling for designing insoles clubfoot.

Furthermore, CBS modelling techniques can create 3D insole images for patients with clubfoot defect [3,8,9,44]. Because the contour curve of the clubfoot patient included the curve, curve border, and roommate, CBS modelling enabled to effectively correct the insole model; the results are shown in Fig. 7. These stages provide a major advantage when editing the generated curve and obtaining a smooth insole surface, as demonstrated previously [[7], [8], [9]] in the design of an insole for diabetic patients with foot deformities. The 3D CAD model shown in Fig. 2 was modified by extending the geometrical scale of the legs according to the geometric tolerances (Fig. 3 and Table 1). For CNC machining, the tool path strategy included three design variations (0, 0.75, and 1.5) that were first simulated using the CAM PowerMill 2016 software. The tolerances set by the scale magnification in the X- and Y-axis directions resulted in modifications to the product design geometry.

Correspondingly, the NC-Code for tool path strategy, spindle speed, step-down, step-over, and cutter diameter was implemented for machining the insoles for clubfoot patients. Raster finishing, optimized constant Z, and step and shallow steps are part of the tool path strategy. According to previous studies [[7], [8], [9]], these machining parameters provide the optimal machining time for ankle-foot orthotics in patients with diabetes. The fastest machining time resulting from the 3D CAD insole model of a clubfoot patient under simulation was validated using the OA L_27_3^6^ matrix.

Fig. 4 depict the stages of the optimisation process using the CAM PowerMill 2016 software based on the provided OA. The simulated machining time was 25% shorter than the actual machining time provided by the CAM-CNC engineer. The total time spent on these experiments was equal to the sum of the time spent on the roughing and finishing processes (when the cutter starts from the zero position until it returns to the tool post). S 3D model of the insole was formed, as shown in Fig. 6. The maximum spindle speed and feed rate (100%) were reported previously [9,18] to influence the tool path approach in PowerMill 2016. It was also reported that the simulation time differed from the actual machining time, including the time required to replace the cutter and set the material on the CNC machine table.

**Fig. 8:**
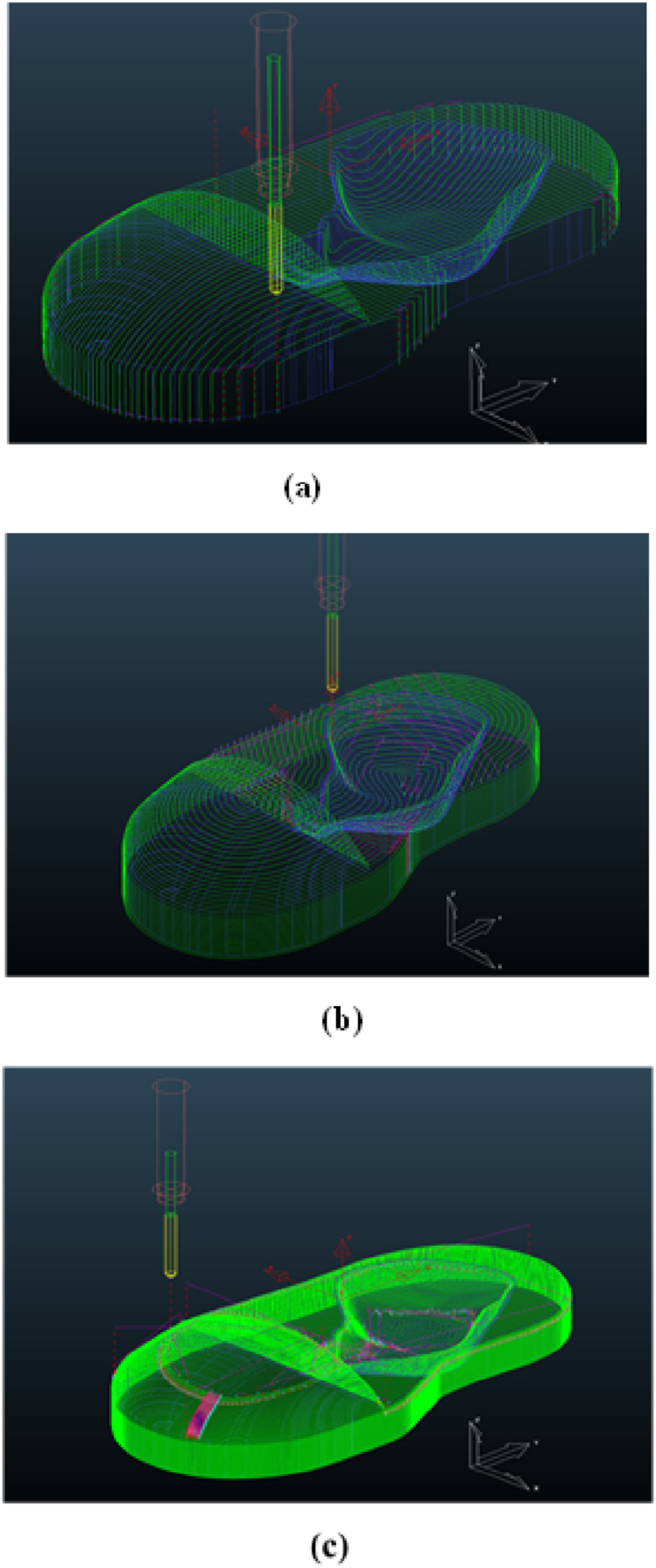
Machining Tool path Strategy: (a) raster finishing, (b) optimized constant Z, (c) step and shallow Finishing.

**Fig. 9:**
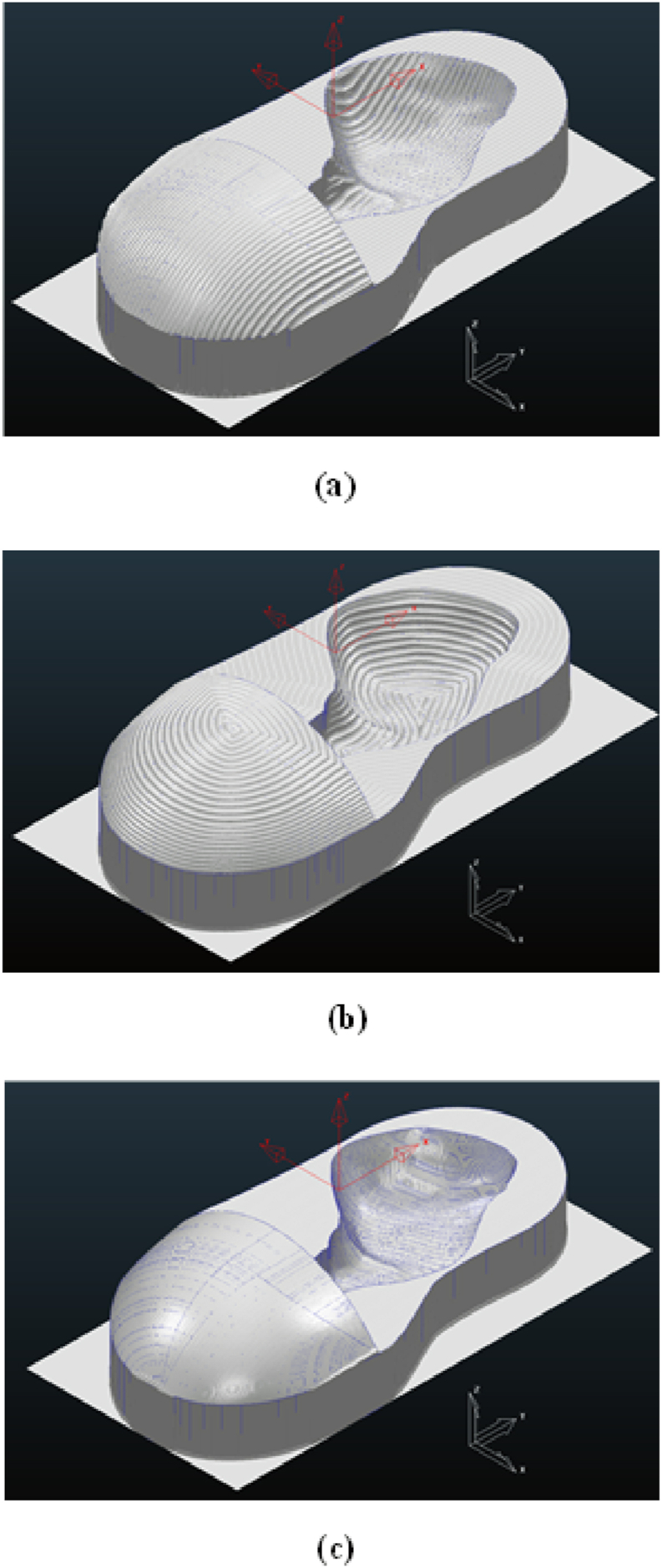
Result Simulation Processed on The PowerMill 2016 Software: (a) raster finishing, (b) optimized constant Z, (c) step and shallow finishing.

During the experimental CNC machining, the faster finishing tool path strategy with a spindle speed of 13,000 rpm, step-down of 3 mm, step-over of 3 mm, and cutter diameter of 6 mm at a tolerance of 0 mm for 150 min provided the fastest machining time. The processing time difference between both tool paths was 56%. This difference may be related to the cutter movement in each step and increased difficulty of implementing the shallow finishing toolpath strategy compared to the raster finishing toolpath strategy (Fig. 7). When compared to the raster finishing toolpath method, the step and shallow movements were closer to the 3D CAD profile of the insole model, according to the physical model of the patient's foot [see Fig. 8, Fig. 9].

### 3.1 Analysis of the S/N ratio with Taguchi method

The S/N Ratio was used to check each component and level that influenced the machining time in the simulation. Table 4, Table 5 report the difference in the maximum and minimum S/N ratios (primary effect). Under optimal machining conditions, the simulation time and fastest machining time were obtained corresponding to the cutting and level parameters for the simulation and real machining at level one for tool path (A), spindle speed (B), and cutter (E); level two for step-over (D) and tolerance (F); and level three for feed depth (G). In terms of machining time, level one machining conditions for tool path (A), spindle speed (B), and cutter (E), level two for step-over (D), tolerance (F), and level three for feed depth (G) can all be attained.

As previously reported by the Taguchi method [41,43,44], the prediction of the machining time of the simulation process and the real time was expressed as a confidence interval (CI) and later compared using the confirmatory value experiment. The results of these comparisons are reported in Table 6, Table 7.

**Table 6:** Comparison between the confirmatory experiment and calculation results using the Taguchi method regarding simulation time.

Eksp.	*Confirmatory experiment* (min)	*Calculated value* (min)	CI	Different	Optimise
*y*_*1*_	160	125.5	34.657	34.600	Success
*y*_*2*_	158	125.5	34.657	32.60	Success
*y*_*3*_	152	125.5	34.657	26.500	Success
*y*_*rata-rata*_	156	125.5	34.657	30.500	Success

**Table 7:** Comparison between confirmatory experiment and calculation results for real-time machining using the Taguchi method.

Eksp.	*Confirmatory experiment* (min)	*Calculated value* (min)	CI	Different	Optimise
*y*_*1*_	233	152.25	90.937	80.75	Success
*y*_*2*_	209	152.25	90.937	56.50	Success
*y*_*3*_	200	152.25	90.937	47.75	Success
*y*_*rata-rata*_	212	152.25	90.937	59.75	Success

The experimental validation results based on the best parameter combinations are shown in Table 6, Table 7. The confidence intervals regarding the minimum machining time for the simulation and real-time machining of the insoles were calculated as 34.657 and 90.937 min, respectively. Validation tests for the responses yielded a 95% confidence interval.

It was reported that the working duration of the novel insole was best accomplished by utilising a raster finishing strategy with minimum spindle speed, step-down, and step-over parameter values [8,41]. Both the simulation and real-world machining times yielded a lower deferent value than the CI value. This indicates that the optimisation approaches used in this study to manufacture new insole boots using CNC machines with patient clubfoot scans were successful.

**Fig. 10:**
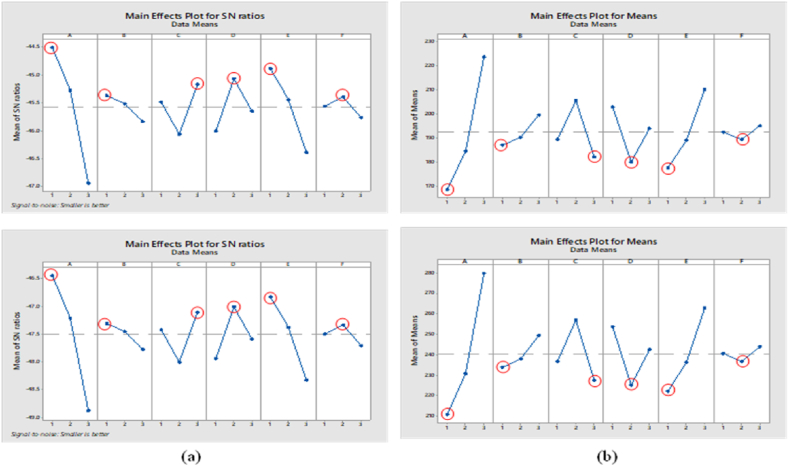
Main effects plots: (a) the average S/N ratio for t_m_, (b) effects of control factors on t_m_.

**Fig. 11:**
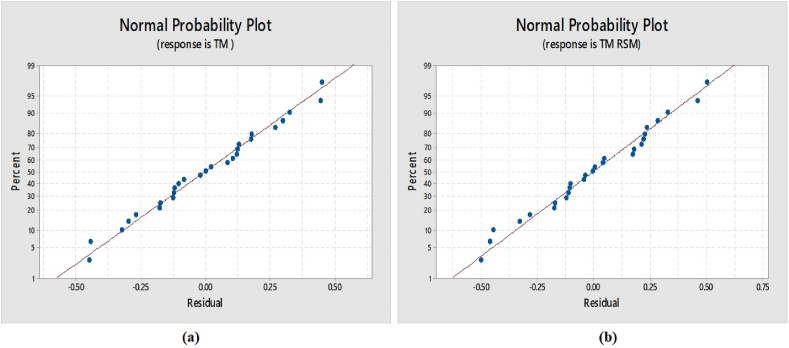
Normality test results: (a) response is TM; (b) response is TM-RSM.

Consequently, A_1_B_1_C_3_D_2_E_1_F_2_ is the ideal cutting parameter condition for the simulation and fastest machining times [see Fig. 10(a and b)]. Accordingly, the achieved optimal conditions, as indicated in Table 4, Table 5, are the tool path strategy for raster finishing (level 1), spindle speed of 10,000 rpm (level 1), step-down of 2.00 mm (level 3), step-over of 3.00 mm (level 2), cutter diameter of 6.00 mm (E1), and insole geometry dimensional tolerance of 0.75 mm (F2). These findings are consistent with the normality test references for the TM and TM-TSM, as shown in Fig. 11(a and b) [33,41,43].

### 3.2 ANOVA in RSM method

The relevance of the regression model and coefficients of individual model parameters (toolpath strategy, spindle speed, feed rate, step-down, cutter diameter, and dimensional tolerance) that affect the simulation and machining times were tested using analysis of variance (ANOVA). Table 8, Table 9 report the ANOVA results for simulation and machining times, respectively. The significance levels of all variables were characterised by the p-value. The regression of both models considered in this investigation yielded a p-value of less than 0.05, indicating that both models significantly affected the response [[38], [39], [40], [41]]. According to Table 8, Table 9, all the modelled parameters have a *p-value* less than 0.05, indicating that all the factors studied significantly impacted the simulation and machining time of insole shoe boots for patients with clubfoot. Because all models have an impact on the findings, a p-value of less than 0.02 is chosen to find the model with the quickest simulation and machining times.

**Table 8:** ANOVA of simulation time for producing insole boots shoes with CAM PowerMill.

	SS	df	MS	F	p
A	**1840.5533**	**1**	**1840.5533**	**2.2946**	**0.0074**
A^2^	312.9283	1	312.9283	0.3901	0.0265
B	1407.4552	1	1407.4552	1.7546	0.0101
B^2^	883.1743	1	883.1743	1.1010	0.0152
C	539.6733	1	539.6733	0.6728	0.0208
C^2^	90.8899	1	90.8899	0.1133	0.0362
D	176.7096	1	176.7096	0.2203	0.0316
D^2^	0.1313	1	0.1313	0.0002	0.0483
E	20.8464	1	20.8464	0.0260	0.0427
E^2^	133.9260	1	133.9260	0.1670	0.0337
F	548.3290	1	548.3290	0.6836	0.0207
F^2^	0.1313	1	0.1313	0.0002	0.0483
AB	**848.9050**	**1**	**848.9050**	**1.0583**	**0.0157**
AC	341.3800	1	341.3800	0.4256	0.0257
AD	40.9050	1	40.9050	0.0510	0.0403
AE	212.2313	1	212.2313	0.2646	0.0301
AF	255.6613	1	255.6613	0.3187	0.0284
BC	**7764.8800**	**1**	**7764.8800**	**9.6803**	**0.0003**
BD	267.1450	1	267.1450	0.3330	0.0280
BE	**955.5913**	**1**	**955.5913**	**1.1913**	**0.0143**
BF	**601.0813**	**1**	**601.0813**	**0.7493**	**0.0196**
CD	50.5000	1	50.5000	0.0630	0.0393
CE	328.3813	1	328.3813	0.4094	0.0261
CF	**2233.2413**	**1**	**2233.2413**	**2.7841**	**0.0057**
DE	**787.9313**	**1**	**787.9313**	**0.9823**	**0.0166**
DF	533.4113	1	533.4113	0.6650	0.0210
EF	341.3800	1	341.3800	0.4256	0.0257
Error	14727.1534	18	818.1747		
Total SS	37558.7387

**Table 9:** ANOVA of real machining time for producing insole boots shoes with CNC milling.

	SS	df	MS	F	p
A	**2904.3378**	**1**	**2904.3378**	**2.2946**	**0.0068**
A^2^	493.7922	1	493.7922	0.3901	0.0246
B	2220.9174	1	2220.9174	1.7546	0.0093
**B**^**2**^	**1393.6260**	**1**	**1393.6260**	**1.1010**	**0.0141**
**C**	**851.5878**	**1**	**851.5878**	**0.6728**	**0.0193**
C^2^	143.4222	1	143.4222	0.1133	0.0336
D	278.8374	1	278.8374	0.2203	0.0293
D^2^	0.2040	1	0.2040	0.0002	0.0448
E	32.8950	1	32.8950	0.0260	0.0396
E^2^	211.3338	1	211.3338	0.1670	0.0312
F	865.2456	1	865.2456	0.6836	0.0192
F^2^	0.2040	1	0.2040	0.0002	0.0448
**AB**	**1339.5456**	**1**	**1339.5456**	**1.0583**	**0.0146**
AC	538.6926	1	538.6926	0.4256	0.0238
AD	64.5456	1	64.5456	0.0510	0.0373
AE	334.8864	1	334.8864	0.2646	0.0279
AF	403.4202	1	403.4202	0.3187	0.0264
BC	**12252.7500**	**1**	**12252.7500**	**9.6803**	**0.0003**
BD	421.5456	1	421.5456	0.3330	0.0260
BE	**1507.8864**	**1**	**1507.8864**	**1.1913**	**0.0133**
BF	**948.4776**	**1**	**948.4776**	**0.7493**	**0.0182**
CD	79.6824	1	79.6824	0.0630	0.0365
CE	518.1702	1	518.1702	0.4094	0.0242
CF	**3523.9776**	**1**	**3523.9776**	**2.7841**	**0.0052**
DE	**1243.3290**	**1**	**1243.3290**	**0.9823**	**0.0153**
DF	**841.7040**	**1**	**841.7040**	**0.6650**	**0.0194**
EF	538.6926	1	538.6926	0.4256	0.0238
Error	23239.0068	18	1291.0548		
Total SS	59266.5696	45			

AB, BC, BE, BF, CF, and DE exhibited p-values less than 0.02 for simulation time, while AB, BC, BE, BF, CF, DE, and DF attained p-values less than 0.02 for machining time. These results demonstrate that by using ideal machining condition settings for CNC milling machines, the shortest simulation and machining time of insole boots shoes for patients with clubfoot defect may be ensured.

### 3.3 RSM-based modeling for surface roughness

RSM was used to model and analyse the dependent and independent variables that could be correlated [36,41,42]. The test results for the insole shoe boots were used to develop the TM mathematical model. Furthermore, various machining models were employed (spindle speed, step-down, tool path strategy, cutter diameter, step-over, and design dimensional tolerance). As a result, the following equations can be used to express the relationship between the simulation time and machining time using the CNC milling parameters considered in this study [Equations (2), (3)]:

(2) $$Simulation\;Time\;Tm\;\left(minutes\right)=46.512+71.924A-2.753A^{2}+1.5.10^{-3}B-1.5.10^{-7}B^{2}+82.1C-0.659C^{2}+1.081D-0.056D^{2}+35.48E-0.45\;E^{2}-15.506F-0.099F^{2}+2.1.10^{-3}AB-2.1.10^{3}AC-0.833CD+1.063CE-7.389CF-2.469DE+5,417DF+2.167.10^{4}EF$$

(3) $$Real\;Time\;Tm\;\left(minutes\right)=46.512+71.924A-2.753A^{2}+1.5.10^{-4}B-1.5.10^{-7}B^{2}+82.1C-0.659C^{2}+1.081D-0.056D^{2}+35.48E-0.451\;E^{2}-15.506F-0.099F^{2}-2.1.10^{-3}AB-2.17.10^{3}AC-1.125AD-1.281AE-3.75AF-4.1.10^{-3}BC-1.1.10^{-3}BD-1.1.10^{-3}BE-2.3.10^{-3}BF+0,833CD+1.063CE-7.389CF-2.469DE+5,417DF+2.167.10^{4}EF$$

The numerical methods used to obtain R^2^ values are then applied in Equations (2), (3). The R^2^ values for the simulation and real production times were 97.80% and 95.62%, respectively. In this example, the R^2^ value was close to 100%, which was ideal for our experiment. Consequently, the abovementioned model can be used to predict the simulation and machining times for specific design parameters.

The processing time for the new insole boots is 263.35 min (simulation time) and 334.86 min (actual time), according to the results of running the V Statistic 5 program on both models (real-time). Using tool path strategy with step and shallow finishing (A) of 3.85, spindle speed (B) of 10,000 rpm, step-down (C) of 2.00 mm, step-over (D) of 3.00 mm, cutter diameter (E) of 6 mm, and dimensional tolerance (F) of 0.75 mm, the two best response values may be obtained. This result agrees with earlier study models developed by Asiltürk and Nesseli (2012) [26], Sarikaya and Güllü (2014) [28], and Anggoro et al. (2021) [41,43].

**Fig. 12:**
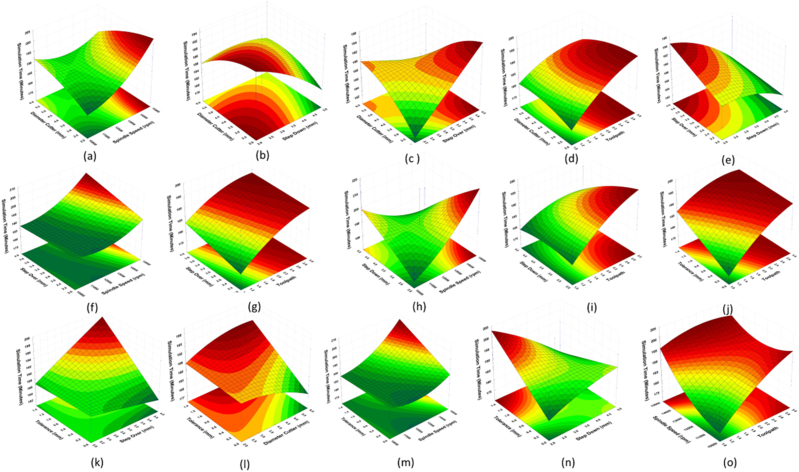
3D plots of simulatino time: (a) Cutter-Spindle Speed; (b) Cutter-Step Down; (c) Cutter – Step Over; (d) Cutter – Toolpath; (e) Step Over – Step Down; (f) Step Over – Spindle Speed; (g) Step Over – Toolpath; (h) Step Down – Spindle Speed; (i) Step Down – Toolpath; (j) Tolerance- Toolpath; (k) Tolerance – Step Over; (l) Tolerance-Cutter; (m) Tolerance - Spindle Speed; (n) Tolerance – Step Down; (o) Spindle Speed – Toopath.

**Fig. 13:**
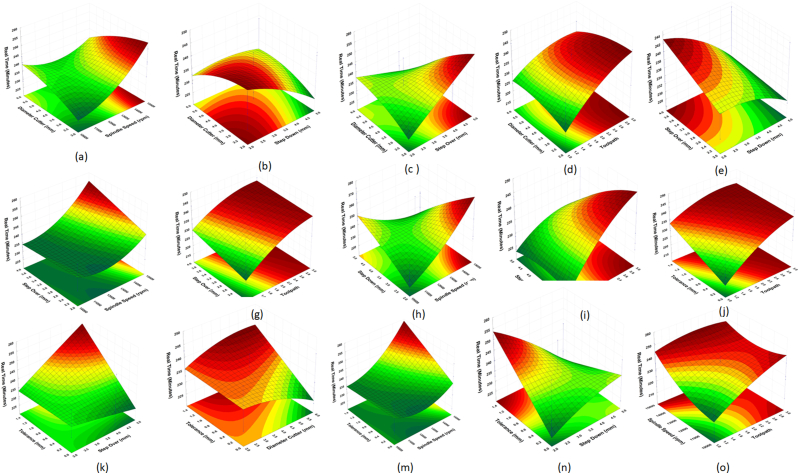
3D plots of real machining time: (a) Cutter-Spindle Speed; (b) Cutter-Step Down; (c) Cutter – Step Over; (d) Cutter – Toolpath; (e) Step Over – Step Down; (f) Step Over – Spindle Speed; (g) Step Over – Toolpath; (h) Step Down – Spindle Speed; (i) Step Down – Toolpath; (j) Tolerance- Toolpath; (k) Tolerance – Step Over; (l) Tolerance-Cutter; (m) Tolerance - Spindle Speed; (n) Tolerance – Step Down; (o) Spindle Speed - Toopath.

A 3D representation of the measured response was created numerically for easily understanding the interaction effect of machining settings on the simulation and machining times (Equations (2), (3)). The 3D plots depict the relationship between the machining process parameters and simulation and machining process times. Fig. 10(a and b) shows the relationship between the parameters and the simulation time, whereas Fig. 12, Fig. 13 show the relationship between the parameters and machining time. Using the best cutter diameter size, high spindle speed, large step-over, deep step-down, and small dimensional tolerance, simulation and machining times were obtained in this study. The cutter rate can be increased by increasing the step-over and spindle speed during the cutting process [33,36]. As the work area of the workpiece being treated is large, using a cutter with a large diameter and deep step-down can accelerate the machining process [41]. Because of adopting the smallest insole width dimensional tolerance, the simulation and runtime were reduced.

Engineers use optimisation techniques to determine the maximum and minimum values of a function [41,42]. The Taguchi response surface methodology (TM-RSM) was employed in this study to demonstrate the statistical significance of the machining parameters and optimal machining data responses obtained during the production of new footwear insoles for clubfoot patients. As reported in Table 11, the TM-RSM optimisation methodology yields a shorter machining time than the TM method. Additionally, its absolute error value was less than that of the TM method, resulting in a percentage improvement of 19.82% for the simulation and 29.19% for the real machining time. This demonstrates that the TM-RSM technique presented in this study is superior to the TM method for identifying the best cutting parameter circumstances [36,41,42].

**Table 10:** Optimal parameters and validation results of the experimental and RSM-predicted values.

Optimal Cutting Parameter Conditions	Toolpath	Spindle Speed (Rpm)	Step down (mm)	Step Over (mm)	Diameter cutter milling (mm)	Tolerance (mm)	Time Machining		Percentage Error (%)
							Exp.	RSM	
							(μm)		
**A**_**1**_**B**_**1**_**C**_**3**_**D**_**2**_**E**_**1**_**F**_**2**_	*Raster Finishing*	10,000	850	0.25	20–35	0.75	**236**	167.35	29.3
							**334**	233.86	30.4

**Table 11:** Comparison of the optimal and predicted results.

Optimisation technique	tm				Absolute error (%)	
	Experiment		Calculation			
	Simulation (min)	Real (min)	Simulation (min)	Real (min)	Simulation	Real
TM	236	334	125.4	152.25	46.86	54.42
TM-RSM	189.22	236.52	236.35	334.86	**19.94**	**29.37**
Percentage improvement (%)	**19.82**	**29.19**	–	–		

### 3.4 Optimisation using desirability function analysis with TM-RSM

**Fig. 14:**
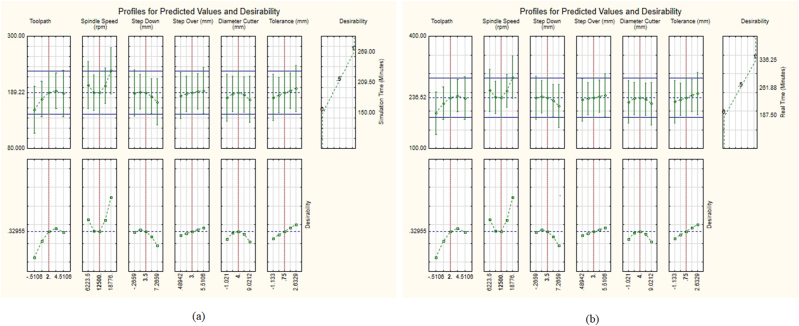
Optimized result of TM-RSM approach: (a) simulation time, (b) real machining time.

The analyzed parameters of the expected response can be transformed into desirability values (dF) using this approach [33]. The dF scale ranges from 0 to 1. It is considered entirely unsatisfactory if the dF value is 0 or close to 0. The value denotes a perfect response to the goal if dF is equal to 1 or close to 1. Because the simulation and machining times can be reached at optimal process settings, dF was chosen based on “the smaller the better” mentality in this study. Fig. 14(a and b) shows the composite desirability (D) and best response according to each control parameter computed using Statistics V5.

Because the ideal minimum time (t_m_) can be obtained after establishing the optimal cutting parameters in a CNC milling machine, the desirability function of t_m_ is chosen based on the quality criterion of “the smaller the better”. The ideal Ta1 value in the model reached 309.22 min, as illustrated in Fig. 13. In this study, the TM-RSM hybrid approach was successfully used to model and optimise the CNC milling of EVA foam for producing orthotic footwear sole. To the best of our knowledge, this is the first study to explore and incorporate experimental and modelling methodologies for producing rubber-based goods using CNC milling. Most existing publications [23,26,30,31,34] have investigated the process parameters in reverse processes. Other studies [28,29,33,35] concentrated on establishing the best CNC milling parameters for applications with a flat surface.

### 3.5 Comfort evaluation

**Fig. 15:**
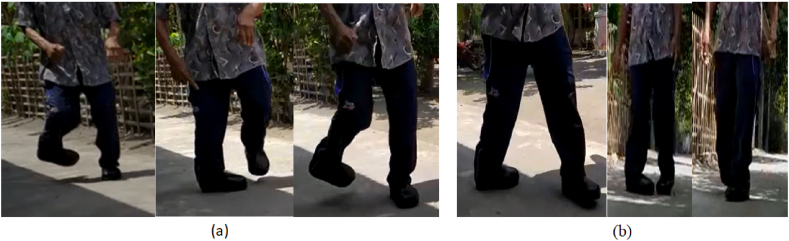
Testing the new boots and insole on adult clubfoot patients: (a) running; (b) walking).

**Fig. 16:**
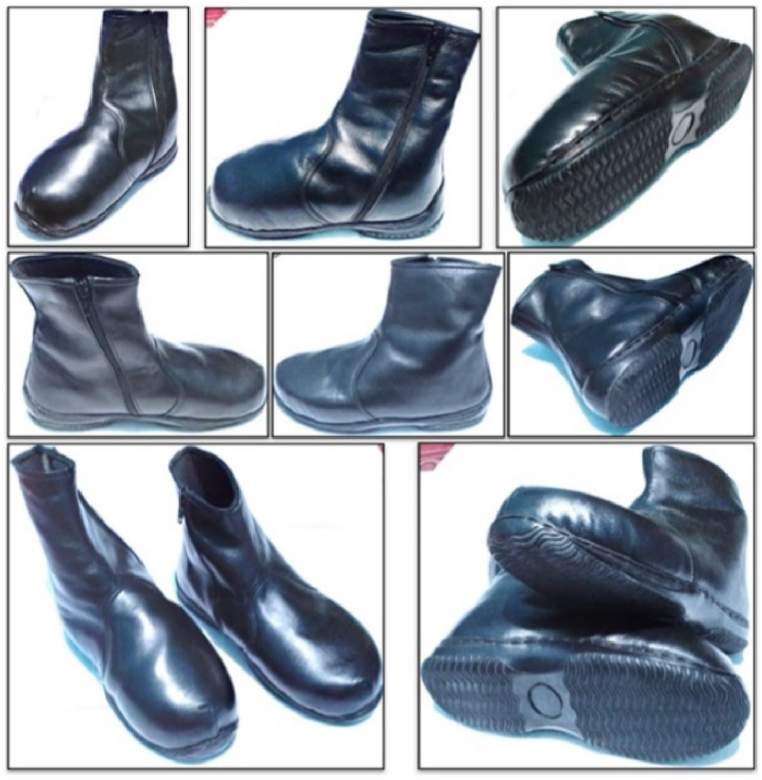
The new boots shoes for adult clubfoot patients.

This study used a sample survey questionnaire to assess the insole and boot designs for clubfoot patients in terms of comfort. Accordingly, we aimed to determine how patients felt about the new boots and insoles that had been designed compared to their old shoes. A previous study adopted a similar questionnaire approach for diabetic and non-diabetic patients [8]. The contents of the questionnaire provided in Table 12 include measurements of the importance, satisfaction, and expectations of patients with clubfoot regarding shoe quality, unrelated to medical clinical studies. Fig. 15(a and b) shows the testing process for the new boots and insoles conducted by researchers on patients with clubfoot defect, which yielded satisfactory results. The shoe wear time was reduced by approximately 85%. The present boots were generally similar to normal boots (Fig. 16).

**Table 12:** The content of the questionnaire for diabetic patients.

No	Attribute	Importance				Satisfaction				Expectation			
		**Prototype of insole boot orthotic hoes**											
1	Good dimension and material	1	2	3	4	1	2	3	4	1	2	3	4
2	Interesting shape	1	2	3	4	1	2	3	4	1	2	3	4
		**Use of boot orthotic hoes**											
3	Lightweight	1	2	3	4	1	2	3	4	1	2	3	4
4	Easy to use initially	1	2	3	4	1	2	3	4	1	2	3	4
5	Comfortable to use	1	2	3	4	1	2	3	4	1	2	3	4
6	Easy to use while walking	1	2	3	4	1	2	3	4	1	2	3	4
7	Easy to use while running	1	2	3	4	1	2	3	4	1	2	3	4
8	Easy to use while standing	1	2	3	4	1	2	3	4	1	2	3	4
9	Protecting the foot while walking from accident	1	2	3	4	1	2	3	4	1	2	3	4
10	Good stability	1	2	3	4	1	2	3	4	1	2	3	4
11	Rigid	1	2	3	4	1	2	3	4	1	2	3	4
12	Durability	1	2	3	4	1	2	3	4	1	2	3	4
13	Easy to duplicate	1	2	3	4	1	2	3	4	1	2	3	4
14	easy for assy by user	1	2	3	4	1	2	3	4	1	2	3	4

It should be emphasised that the patient had worn this boot for approximately six months. Table 10 displays the findings of the questionnaire. Patients 1 and 2 expressed high levels of 3.9–4.0 satisfaction (very satisfied). In terms of expectations, patients 1 and 2 scored 3.8 (extremely satisfied) and 3.7 (satisfied), respectively. This suggests that each user’s level of satisfaction was higher than their expectations. In other words, patients were highly pleased with the comfort and performance of their shoes.

Table 13 reports that after six months of testing the boots, the satisfaction rating for patients with clubfoot defect is approximately 3.93 (extremely satisfied). The score for expectancy was approximately 3.43 (satisfied). This demonstrates that the level of patient satisfaction achieved in this study is higher than the expectation levels of these patients. In other words, the patients with clubfoot defect considered in this study were significantly satisfied with the shoe comfort and performance. This is demonstrated by the patient wearing the boots daily.

**Table 13:** The results of the questionnaire conducted on the patient with clubfoot defect.

No	Attribute	Importance	Satisfaction	Expectation
**Prototype of insole boots orthotic shoe**				
1	Good dimension and material	4	4	3
2	Interesting shape	4	4	3
**Use of boot shoes orthotic**				
**Use of OIS (orthotic insole shoe)**				
3	Lightweight	4	4	3
4	Easy to use initially	4	4	3
5	Comfortable to use	4	4	4
6	Easy to use while walking	4	4	4
7	Easy to use while running	3	3	3
8	Easy to use while standing	4	4	4
9	Protecting the foot while walking from accident	4	4	3
10	Good stability	4	4	3
11	Rigid	3	4	4
12	Durability	4	4	4
13	Easy to duplicate	4	4	3
14	easy for assy by user	4	4	4

## 4 Conclusion

a) Based on the analysis, the optimal parameters are A_1_B_1_C_3_D_2_E_1_F_2_ with A (tool path strategy of raster finishing or set #1), B (spindle speed of 10,000 rpm or set #1), C (step-down of 850 mm or set #3), D (step-over of 0.25 mm or set #2), E (cutter diameter of 20–35 mm or set # 1), and F (dimensional tolerance of 0.75 mm or set # 2).
b) A comparison of the optimisation results with the Taguchi method (TM) and TM-RSM method underlines yields the differences in the obtained results. The TM method simulation machining times were 236 and 125.4 min, and real machining times were 334 and 152.25 min with error values of 46.86% and 54.42%, respectively. Meanwhile, with the TM-RSM method, simulation machining times of 189.22 and 236.35 min were obtained, while the real machining times were 236.52 and 334.86 min with error values of 19.94% and 29.37%, respectively. Therefore, there was an improvement of 19.82% (simulation time) and 29.19% (real-time) between both methods.
c) The production time of orthotic boots insoles for clubfoot patients were predicted in a model-based manner using the Taguchi method, response surface modeling, and desirability function (TM-RSM). The CNC milling machine successfully generated a 3D plot surface of the processing time, cutting parameter condition, and product insole boot shoes for patients with clubfoot defect.
d) The testing results of boots with insoles developed in this study indicate that the level of patient satisfaction was higher than expected for these individuals. The satisfaction score was 3.93 (scale 1–4), which was higher than the expected score of 3.43 (scale 1–4). In other words, the patients with clubfeet considered in this study were significantly satisfied with the shoe comfort and performance. This was demonstrated by the patient's continued daily use of these boots.
e) In the future, to improve the quality of life of patients, the effect of shoe utilisation should be explored to provide more feedback to designers, engineers, doctors of orthotics, and prosthetics makers worldwide, as well as in the Indonesian community.

## Funding statement

This research was funded by Ministry of Education, Culture, Research, and Technology Directorate GEneral of Higher Education; Director of Resources Directorate General of Higher Education, Research, and Technology, Fiscal Year 2021; Grand Number: 2817/E4.1/KK.04.05/2021; 257-93/UN7.P4.3/PP/2021; 257-73/UN7.P4.3/PP/2021.

## Author contribution statement

Paulus Wisnu Anggoro: Conceived and designed the experiments; Analyzed and interpreted the data; Wrote the paper.

B. Bawono, Lucia Ratnasari, Pniel Kevin Fergiawan: Performed the experiments; Contributed reagents, materials, analysis tools or data.

Djoko Budiyanto Setyohadi, J. Jamari: Analyzed and interpreted the data.

M. Tauviqirrahman: Conceived and designed the experiments; Wrote the paper.

A.P. Bayuseno: Contributed reagents, materials, analysis tools or data; Wrote the paper.

## Data availability statement

Data included in article/supp. material/referenced in article.

## Declaration of competing interest

The authors declare that they have no known competing financial interests or personal relationships that could have appeared to influence the work reported in this paper.
